# Optical Detection of Ketoprofen by Its Electropolymerization on an Indium Tin Oxide-Coated Optical Fiber Probe

**DOI:** 10.3390/s18051361

**Published:** 2018-04-27

**Authors:** Robert Bogdanowicz, Paweł Niedziałkowski, Michał Sobaszek, Dariusz Burnat, Wioleta Białobrzeska, Zofia Cebula, Petr Sezemsky, Marcin Koba, Vitezslav Stranak, Tadeusz Ossowski, Mateusz Śmietana

**Affiliations:** 1 Faculty of Electronics, Telecommunications and Informatics, Gdansk University of Technology, Narutowicza 11/12, 80-233 Gdansk, Poland; rbogdan@eti.pg.gda.pl (R.B.); micsobas@pg.edu.pl (M.S.); 2 Department of Analytical Chemistry, Faculty of Chemistry, University of Gdansk, Wita Stwosza 63, 80-308 Gdansk, Poland; pawel.niedzialkowski@ug.edu.pl (P.N.); wioleta.bialobrzeska@phdstud.ug.edu.pl (W.B.); zofia.jelinska@phdstud.ug.edu.pl (Z.C.); tadeusz.ossowski@ug.edu.pl (T.O.); 3 Institute of Microelectronics and Optoelectronics, Warsaw University of Technology, Koszykowa 75, 00-662 Warszawa, Poland; drkbrt@o2.pl (D.B.); marcinkoba@gmail.com (M.K.); 4 Institute of Physics and Biophysics, Faculty of Science, University of South Bohemia, Branisovska 1760, 370 05 Ceske Budejovice, Czech Republic; petr.sezemsky@gmail.com (P.S.); stranv00@centrum.cz (V.S.); 5 National Institute of Telecommunications, Szachowa 1, 04-894 Warszawa, Poland

**Keywords:** ketoprofen, anti-inflammatory drug, drug analysis, optical fiber sensor, reactive magnetron sputtering thin film, indium tin oxide (ITO), lossy-mode resonance (LMR), electrochemistry, electropolymerization

## Abstract

In this work an application of optical fiber sensors for real-time optical monitoring of electrochemical deposition of ketoprofen during its anodic oxidation is discussed. The sensors were fabricated by reactive magnetron sputtering of indium tin oxide (ITO) on a 2.5 cm-long core of polymer-clad silica fibers. ITO tuned in optical properties and thickness allows for achieving a lossy-mode resonance (LMR) phenomenon and it can be simultaneously applied as an electrode in an electrochemical setup. The ITO-LMR electrode allows for optical monitoring of changes occurring at the electrode during electrochemical processing. The studies have shown that the ITO-LMR sensor’s spectral response strongly depends on electrochemical modification of its surface by ketoprofen. The effect can be applied for real-time detection of ketoprofen. The obtained sensitivities reached over 1400 nm/M (nm·mg^−1^·L) and 16,400 a.u./M (a.u.·mg^−1^·L) for resonance wavelength and transmission shifts, respectively. The proposed method is a valuable alternative for the analysis of ketoprofen within the concentration range of 0.25–250 μg mL^−1^, and allows for its determination at therapeutic and toxic levels. The proposed novel sensing approach provides a promising strategy for both optical and electrochemical detection of electrochemical modifications of ITO or its surface by various compounds.

## 1. Introduction

Demand for nonprescription drugs, such as 2-(3-benzoylphenyl)-propanoic acid (ketoprofen, KP) is expected to increase in the near future. KP is a nonsteroidal anti-inflammatory drug, widely used for the treatment of various kinds of pains, rheumatoid arthritis and osteoarthritis [1]. KP exhibits analgesic and antipyretic activity, which is mainly caused by the inhibition of prostaglandin synthesis by inhibiting cyclooxygenase [2]. The widespread and growing volume of human and veterinary prescriptions needs to be followed by the development of analytical techniques allowing for detection of KP traces in various biofluids or sludge water.

Several methods have already been reported for quantitative determination of KP, including liquid chromatography-mass spectrometry [3], UV-fluorescence [4], ion chromatography [5], flow injection with chemiluminescence [6], or electrochemical detection [7]. Both chromatographic and non-chromatographic techniques usually require rigorous sample preparation and expensive extraction methods (including solid-phase extraction) when real samples are considered. Mass spectrometry in turn requires analyte signal suppression or enhancement during electrospray ionization, especially for analysis of multi-compound samples. Application of all these methods is time-consuming and requires expensive and highly specialized setups which are only available in well-equipped research laboratories. Among other techniques, fluorescence at porous SnO_2_ nanoparticles was applied for KP detection using combined ion chromatography with photodetection. The limit of detection (LOD) in human serum, urine, and canal water samples was 0.1 μg/kg, 0.5 μg/kg, and 0.39 μg/kg, respectively [5]. However, photometric UV and fluorescence-based methods commonly suffer from low sensitivity and selectivity, while the latter require specific chemicals or compounds (e.g., nanoparticles) in the detection procedure.

Electrochemical studies of KP have already been performed using the polarographic method [8,9] or simultaneously cyclic voltammetry and coulometry techniques at the mercury dropping electrode surface [8,10]. Kormosh et al. [11] developed ion-selective electrodes for potentiometric analysis of KP in piroxicam based on Rhodamine 6G in a membrane plasticizer. The measurements of KP using direct current stripping voltammetry as well as spectrophotometric methods were also reported by Emara et al. [9]. With dropping mercury electrode and using different supporting electrolytes at different pH values it was possible to reach a LOD as low as 5.08 × 10^−4^ ng mL^−1^. A similar electrode was utilized by Ghoneim and Tawfik [10], and in a Britton-Robinson buffer (pH 2.0) the LOD was 0.10 ng mL^–1^. Next, a setup containing glassy carbon (GC) electrode with multiwalled carbon nanotubes/ionic liquid/chitosan composite for covalent immobilization of the ibuprofen by specific aptamer was proposed [7].

It can be concluded that the electrochemical methods offer reliability and accuracy, enabling development of simple, rapid, and cost-effective approaches for the detection of electroactive compounds. However, electrodes for KP detection usually need complex pre-treatment in order to reach high repeatability and environmental stability. Furthermore, KP shows poor solubility in water, a tendency to adsorb and block electrodes, and a rapid metabolization to by-products [12]. Thus, conventional electrochemical assay procedures are not well-suited for this specific application.

To overcome the limitations listed above, we propose a novel approach for KP detection, where together with an electrochemical method an indium tin oxide (ITO)-coated optical fiber sensor is used. ITO is known for its high optical transparency and low electrical resistivity. Moreover, thanks to its band-gap, it is a good candidate for an electrochemical electrode, and it can be used for optical measurements as well. Contrary to other transparent electrode materials, such as boron-doped diamond, thin ITO films can be deposited at a relatively low temperature on various substrates and shapes [13]. Plasma-assisted deposition is often used for obtaining high quality ITO films. An ITO overlay was applied as a standard working electrode, where KP was electropolymerized with the cyclic voltammetry technique. Thanks to the adjustment of both ITO’s electrochemical and optical properties, lossy-mode resonance (LMR) effect in optical fiber sensor can also be applied for KP detection. LMR is a thin-film-based optical effect, which takes place when a certain relation between electric permittivity of the film, substrate, and external medium is fulfilled, namely the real part of the film’s electric permittivity must be positive and higher in magnitude than both the thin film’s permittivity imaginary part and the permittivity of the analyte [14]. Any variation in optical properties of the analyte, especially its refractive index (RI), has an influence on resonance conditions and can thus be detected. Since in visible spectral range ITO shows relatively high RI (n_D_ ~ 2 RIU) and non-zero extinction coefficient (corresponding to optical absorption), it has already been successfully applied in LMR-based sensing devices [15]. There have been reported applications of other thin films supporting the LMR effect such as diamond-like carbon [16] , SiN*_x_* [17], TiO_2_ [18], and polymers [19], but among these materials only ITO offers low electrical resistivity and can be applied as an electrode material. Until now both the electrical conductivity of ITO and supported by its thin film LMR effect have been applied only with the purpose of inducing a high voltage change in properties of an electro-optic material deposited on ITO surface [20], and for electropolymerization of a chemical compound on the ITO surface [21]. As an alternative to the conventional assay procedures, the developed opto-electrochemical probe can offer a KP detection method free from pre-treatment of the electrode’s surface, as well as prolonged analysis time and sophisticated experimental setup.

The application of opto-electrochemical probes is a novel approach. To the best of our knowledge, in this paper we discuss for the first time the application of LMR phenomenon at the ITO-coated optical fiber for electrochemically-induced KP detection. The developed opto-electrochemical probe offers capability for label-free KP detection with no need of the electrode’s surface pre-treatment. Additionally, the GC electrode has been modified by KP for reference.

## 2. Materials and Methods

KP (2-(3-benzoylphenyl) propionic acid) of purity greater than 98% was obtained from Cayman Company (Ann Arbor, MI, USA) and used without any further purification. A KP solution with a concentration of 2 mM was prepared in a 0.1 M phosphate buffer saline (pH = 7.0). Na_2_SO_4_ and K_3_[Fe(CN)_6_] were purchased from POCh (Gliwice, Poland).

### 2.1. ITO Optical Probe Fabrication and Testing

The LMR structures were fabricated using approx. 15 cm-long polymer-clad silica fiber samples of 400/840 μm core/cladding diameter, where 2.5 cm of polymer cladding was removed in the fiber central section [22]. Next, the electrically conductive and optically transparent ITO films were deposited by reactive magnetron sputtering of ITO target (In_2_O_3_-SnO_2_—90/10 wt % and purity of 99.99%). The magnetron, whose axis was perpendicular to the substrate, was supplied by a Cito1310 (13.56 MHz, 300 W) RF source (Comet AG, Flamatt, Switzerland). The experiments were carried out at pressure *p* = 1.0 Pa in a reactive N_2_/Ar atmosphere, gas flows were 15 and 0.5–1.0 sccm for Ar and N_2_, respectively. The overlays were deposited on fibers rotated in the chamber during the process. Simultaneously, Si wafers and glass slides were also coated for reference. Both of the end-faces of the fiber sample were mechanically polished before the optical testing. 

To determine RI sensitivity of the fabricated ITO-LMR devices, they were investigated in the air and mixtures of water/glycerin with n_D_ = 1.33–1.45 RIU. The RI of the mixtures was measured using an AR200 automatic digital refractometer (Reichert Inc., Buffalo, NY, USA). The optical transmission of the ITO-LMR structure was interrogated in the range of λ = 350–1050 nm using an HL-2000 white light source (Ocean Optics Inc., Largo, FL, USA) and an Ocean Optics USB4000 spectrometer. The optical transmission (*T*) in the specified spectral range was detected as counts in specified integration time (up to 100 ms). The temperature of the solutions was stabilized at 25 °C to avoid thermal shift of the RI.

### 2.2. Electrochemical Setup and Electropolymerization of KP

**Figure 1:**
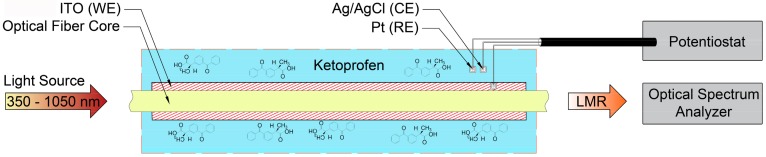
The schematic representation of the experimental setup with ITO-LMR probe used for combined optical and electrochemical KP detection. The electrodes were denoted as working (WE), reference (RE), and counter (CE).

Cyclic voltammetry measurements were performed with a PGSTAT204 potentiostat/galvanostat (Metrohm, Herisau, Switzerland) controlled by Nova 1.1 software, and using the ITO-LMR probe as a working electrode (WE), a platinum wire as counter electrode (CE), and an Ag/AgCl/0.1 M KCl as a reference electrode (REF). The ITO-LMR working electrode was electrochemically processed in 0.1 M phosphate buffer saline containing from 1 × 10^−6^ to 1 × 10^−3^ M of KP at scan rate 50 mV·s^−1^ for 6 cycles. The process allowed for anodic electrooxidation of KP in the potential ranging from 0.3 to 2.0 V vs. Ag/AgCl/0.1 M KCl electrode. The reference GC and ITO electrodes were processed under the same conditions as the ITO-LMR electrode, but for the GC electrode the modification took 10 cycles. Next, the electrodes were washed in water and methanol, and dried under a stream of air. The electrode examinations before and after modification with cyclic voltammetry were performed in 5 mM of K_3_[Fe(CN)_6_] in 0.5 M Na_2_SO_4_ solution at scan rate of 100 mV·s^−1^. The setup used in this experiment is schematically shown in Figure 1.

### 2.3. X-ray Photoelectron Spectroscopy Surface Studies

X-ray Photoelectron Spectroscopy (XPS) studies were carried out using an ESCA300 XPS setup (Scienta Omicron GmbH, Taunusstein, Germany) with a high resolution spectrometer equipped with a monochromatic Kα source. Measurements were done at 10 eV pass energy and 0.05 eV energy step size. A flood gun was used for charge compensation purpose. Finally, the calibration of XPS spectra was performed for carbon peak C1s at 284.6 eV [23,24].

## 3. Results and Discussion

### 3.1. The RI Sensitivity of the ITO-LMR Probe

**Figure 2:**
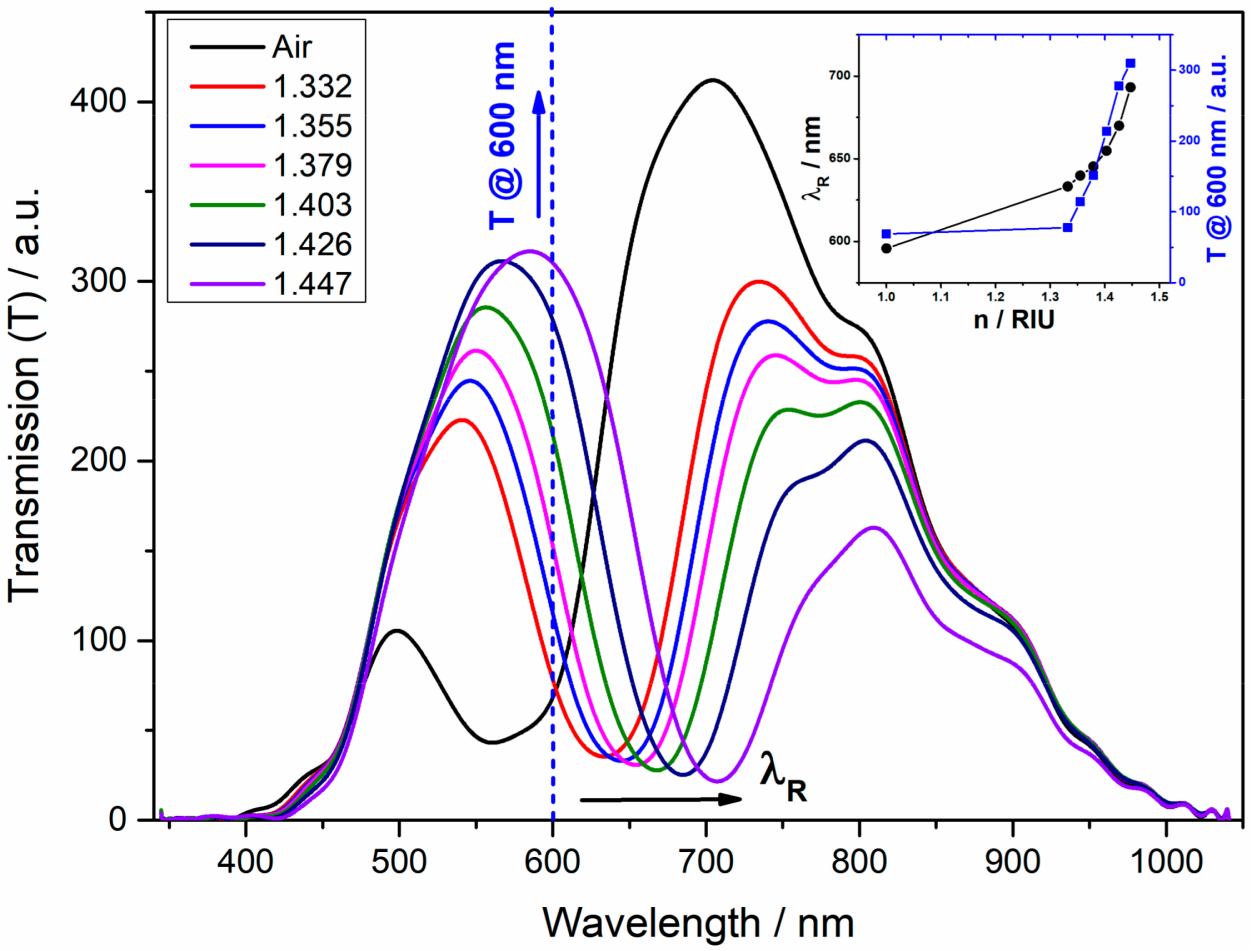
Spectral response of ITO-LMR probe to changes in external RI (n). The changes of resonance wavelength (λ_R_) and transmission (T) at λ = 600 nm are shown in the inset.

First, the optical probes were studied in an optical setup only. This part of the experiment was done in order to estimate the sensitivity of the device to changes of optical properties at the ITO surface. The probes were installed in a setup allowing one to record the transmission spectra, while the sensor was consecutively immersed in different RI solutions. In Figure 2 a well-defined resonance can be seen that experiences a shift towards higher wavelengths when the external RI increases. It is worth noting that the applied ITO coatings provide relatively narrow resonance. The full width at half maximum (FWHM) obtained for the resonances is approx. 110 nm. When the probe is immersed in the higher RI, the FWHM of the resonance slightly increases. Based on the obtained results, two ways of sensor interrogation can be selected, namely tracking of resonance wavelength (λ_R_) or monitoring transmission at discrete wavelength, the most effectively chosen at the resonance slope. In the case of this experiment, the transmission was monitored at λ = 600 nm (T_600_). Both the λ_R_ and T_600_ were plotted vs. RI in the inset of Figure 2. The shift is positive for both of the interrogation schema, but for tracking λ_R_ the dependence is less linear (the sensitivity increases with RI) than for the T_600_. 

The measurements of reference Si samples allowed to estimate the thickness of the coating to 260 nm. According to theoretical studies [25], a low order LMR may be observed for such thickness of ITO. It is known that low order LMRs offer the highest sensitivity to changes in external RI [14], as well as changes in properties of a layer formed on the ITO surface [26].

The standard commercial ITO electrodes undergo thermal annealing to decrease both optical absorption and electrical resistivity, most likely due to the crystallization processes [27]. The LMR-satisfying properties of non-annealed, as fabricated ITO films are attributed to unique advantages of the applied discharge during deposition process. Our previous research clearly showed that optimization of the deposition pressure 0.5 Pa < p < 1.0 Pa induces collisions of the sputtered particles [28,29]. The application of magnetron sputtering is advantageous and allows to tailor the optical and electrical properties of the deposited ITO films with no additional post-deposition annealing as it is often required in case of other deposition methods [30].

### 3.2. Electrodeposition of KP on GC, ITO and ITO-LMR Electrodes

**Scheme 1:**
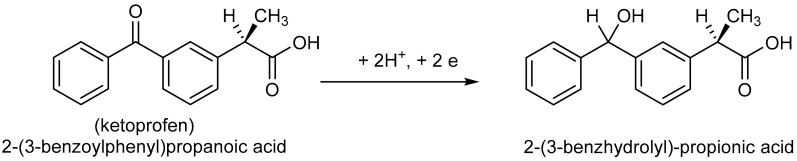
Chemical structure of KP and mechanism of its electrochemical reduction.

The electrochemical deposition of KP on GC, ITO and ITO-LMR electrodes was made by anodic oxidation. The redox behavior of KP molecule at the GC and ITO electrode has not been reported yet. However, the anodic oxidation of KP was observed at the boron doped diamond electrode. The current peak measured for this electrode during the electrodeposition processes is associated with the oxidation of carboxyl group in KP [31]. Moreover, the mechanism of the electrochemical reduction of KP have been until now examined only at the mercury electrode and requires transfer of two electrons. This reduction leads to formation of 2-(3-benzhydrolyl)-propionic acid [8] (Scheme 1). The mechanism of reduction of KP—benzophenone-3 has been described elsewhere [32].

Anodic oxidation by electron transfer leads to deactivation of electrode surface and its modification by adsorption of oxidized polymeric products of the reaction. This effect has been used in this work for modification of different types of electrodes, i.e., GC, ITO, and ITO-LMR. It must be emphasized that only ITO-LMR electrode allows for simultaneous optical and electrochemical monitoring of the modification processes by KP.

**Figure 3:**
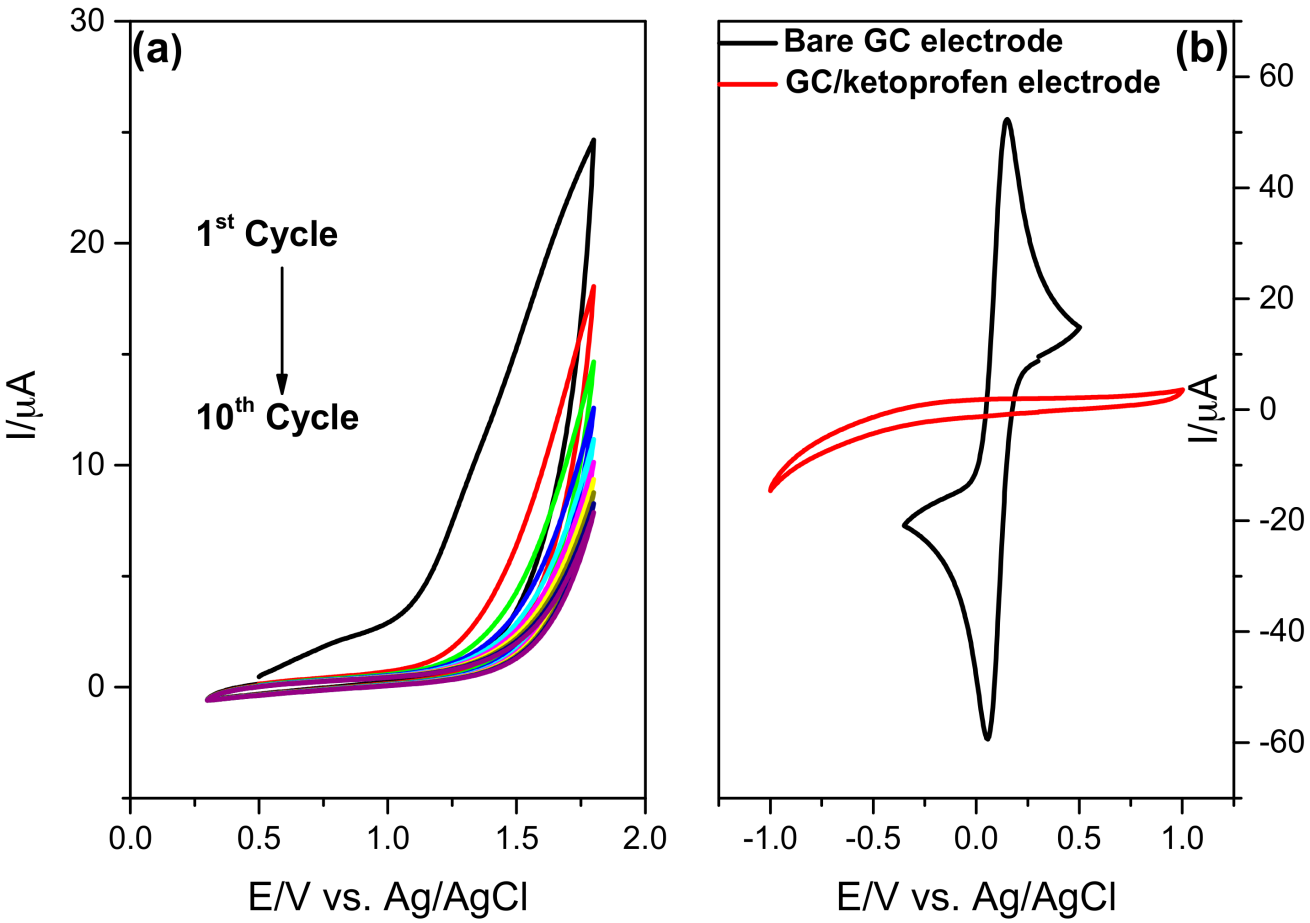
Cyclic voltammetry curves recorded for (**a**) GC electrode in 0.1 M phosphate buffer saline containing 2 mM of KP for 10 cycles, scan rate of 50 mV·s^−1^; and (**b**) bare GC and GC/KP electrode in 0.5 M Na_2_SO_4_ containing of 5 mM [Fe(CN)_6_]^3−/4−^. The scan rate was set to 100 mV·s^−1^.

The cyclic voltammetry is a very valuable and convenient tool to monitor the electrode surface properties before and after each step of modification [33]. The electrochemical responses of the bare GC, ITO and ITO-LMR electrodes were investigated in a solution of 0.5 M Na_2_SO_4_ containing 5 mM [Fe(CN)_6_]^3−/4−^. The comparison of cyclic voltammograms of modified and bare electrodes is presented in Figure 3. For the bare GC electrode, well-defined reversible redox peaks corresponding to one-electron reversible reaction and the peak to peak separation value of 95 mV were reported [34]. This redox reaction of KP completely blocked the GC electrode after 10 scans. The current peak observed in the first scan (Figure 3a) can be attributed to the oxidation of carboxyl group of the KP [35]. The absence of any current for the GC electrode cycled in presence of KP suggests that KP-based layer was formed on the GC surface (Figure 3b). Polymerized KP film the most likely prevents from the penetration of the electroactive substance towards the electrode surface [36]. This phenomenon has been commonly observed for a series of compounds and different electrode material [37,38].

**Figure 4:**
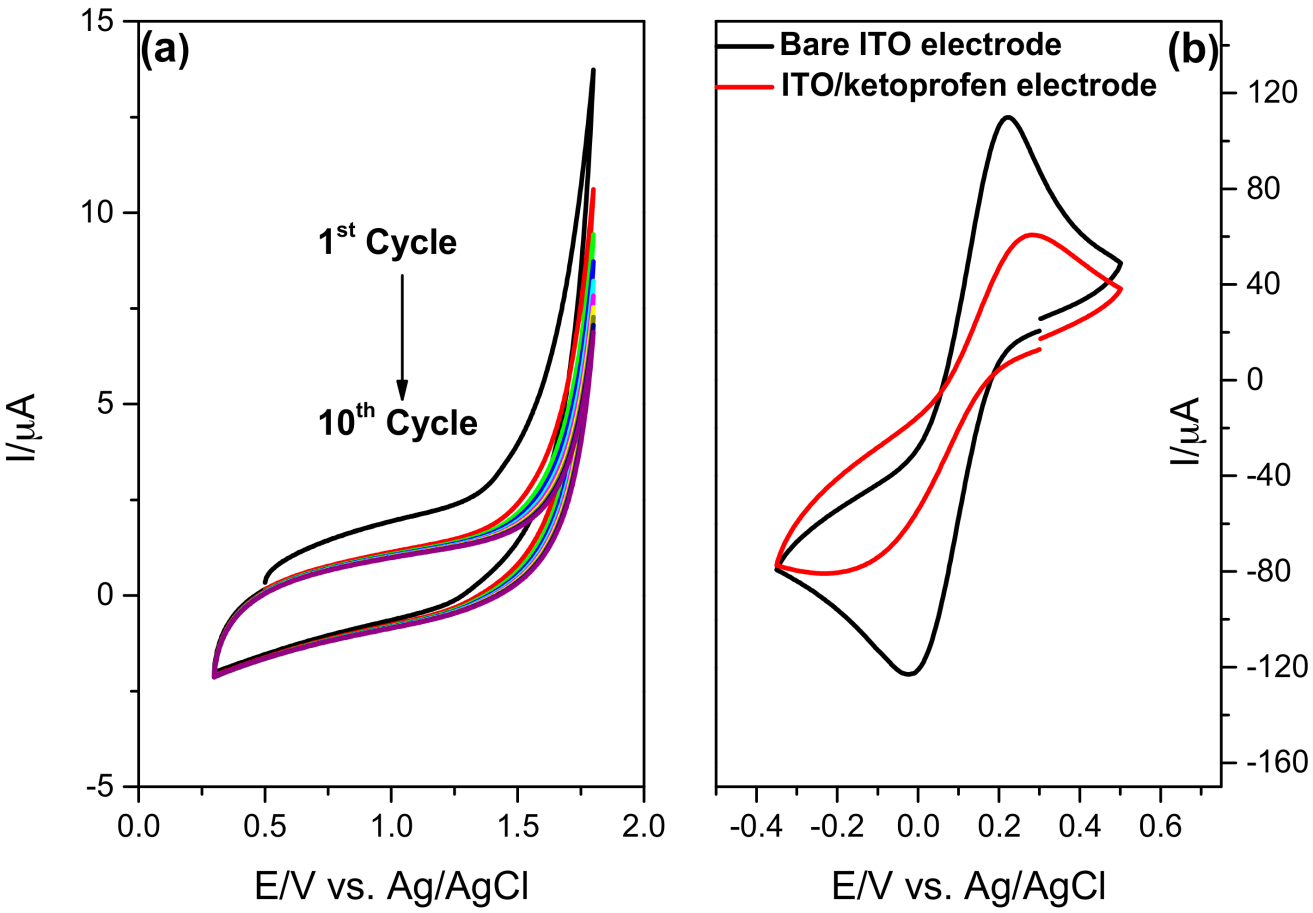
Cyclic voltammetry curves recorded for (**a**) ITO electrode in 0.1 M phosphate buffer saline containing 2 mM of KP (10 cycles, scan rate of 50 mV·s^−1^) and (**b**) ITO and ITO/KP electrode in 0.5 M Na_2_SO_4_ containing 5 mM [Fe(CN)_6_]^3−/4−^, scan rate 100 mV·s^−1^.

Next, a reference ITO electrode deposited on a glass slide underwent a similar modification procedure. In Figure 4a the cyclic voltammetry curves recorded for ITO electrode in 0.1 M phosphate buffer saline containing 2 mM of KP in 10 cycles are shown. For the bare ITO electrode a redox response with peak to peak potential separation reached 245 mV. Significant differences between the electrochemical response for bare and modified electrodes recorded in 0.5 M Na_2_SO_4_ solution containing 5 mM [Fe(CN)_6_]^3−/4−^ were observed. The decrease in peak current and increase in peak to peak potential separation of up to 511 mV, indicate that the modification of the electrode surface by KP was effective (Figure 4b). This phenomenon also suggests that the surface of the ITO electrode was blocked by KP, which is observed in the disappearance of the anodic and cathodic peak [39].

**Figure 5:**
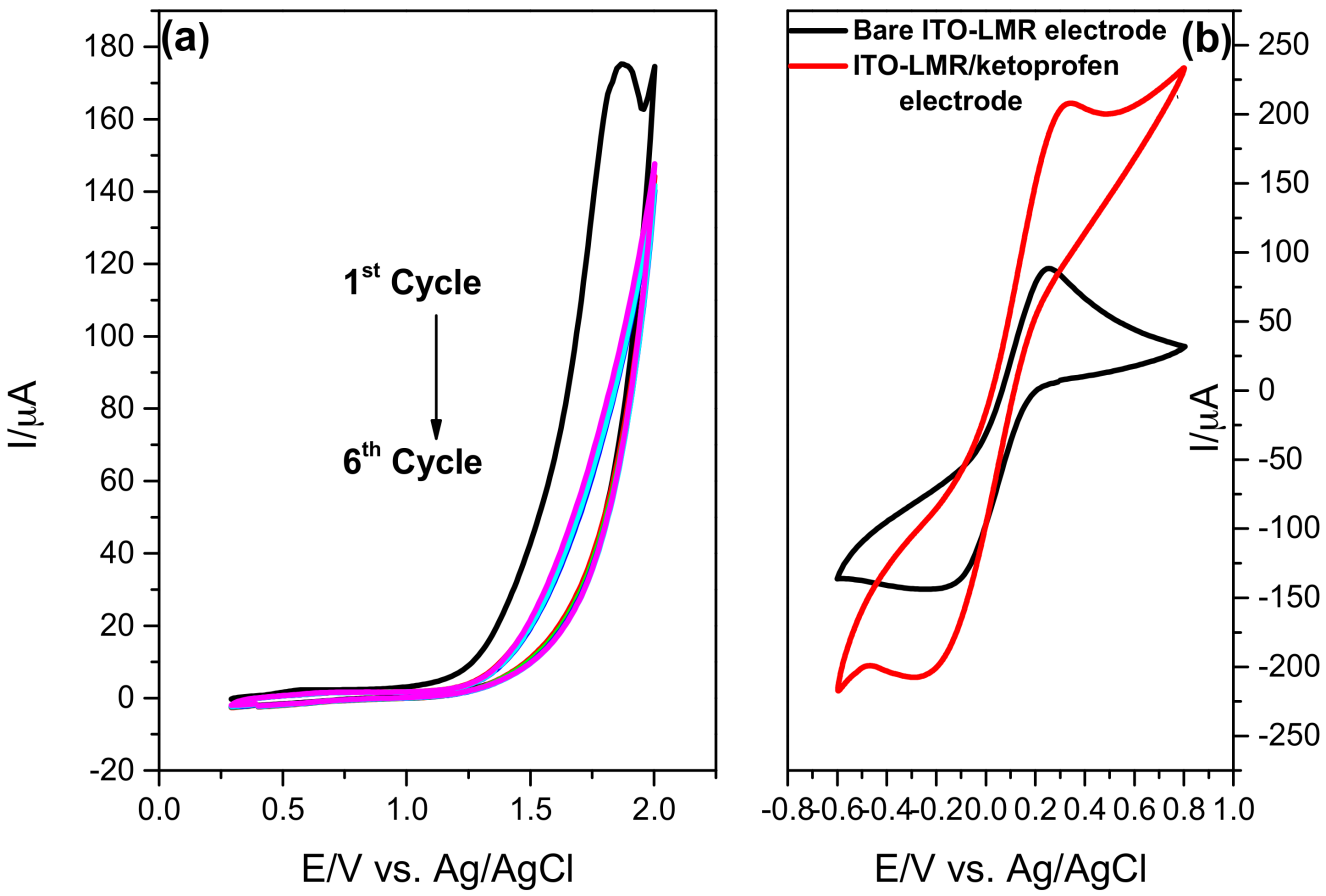
Cyclic voltammetry curves recorded for (**a**) ITO-LMR electrode in 0.1 M phosphate buffer saline containing 2 mM of KP for 6 cycles at scan rate of 50 mV·s^−1^; and (**b**) bare for and KP-modified ITO-LMR in 0.5 M Na_2_SO_4_ containing 5 mM [Fe(CN)_6_]^3−/4−^, scan rate 100 mV·s^−1^.

The ITO-LMR probe was coated with KP during only six anodic oxidation cycles in the potential range from 0.3 to 2.0 V and with a scan rate of 50 mV·s^−1^ (Figure 5a). After this process, the electrode was extensively washed with water and methanol. It is worth noting that for the modification by KP of GC and ITO electrodes, 10 cycles were applied. In the case of ITO-LMR electrode, six cycles were enough to completely cover the electrode. In Figure 5b the cyclic voltammetry response to 0.5 M Na_2_SO_4_ containing 5 mM [Fe(CN)_6_]^3−/4−^ is shown for bare and KP-modified ITO-LMR electrode. Bare and modified ITO-LMR electrodes show redox responses with peak to peak potential separation reaching 419 mV and 628 mV, respectively. Moreover, for the KP-modified ITO-LMR electrode the redox current peaks significantly increased. This effect suggests that the electrode has a more developed active surface area than the one before modification [40]. This was only observed for anodic oxidation of KP on the ITO-LMR electrode. In the other cases, namely reference ITO and GC electrodes, the current peaks for redox couple decreased significantly as a result of modification by KP.

In Table 1 are summarized the electrochemical results obtained for the samples before and after KP modification. The peak splitting difference between bare ITO and ITO-LMR electrodes, i.e., ΔE reaching 245 mV and 419 mV, respectively, can originate from two effects. First, the ITO deposition on cylindrical shape, such as optical fiber, is more challenging than on a flat surface and has an impact on size, crystallinity and morphology of the electrode active surface. Second, the KP modifies the shape of the cyclic voltammetry curves and the peak to peak separations ΔE from 511 to 628 mV for ITO and ITO-LMR electrode, respectively. The diffusion of electrons through the KP is disturbed, revealing slightly reduced electrocatalytic activities, which can be attributed to its structural features and electrochemical properties.

### 3.3. XPS Studies of KP-Modified ITO Surface

**Figure 6:**
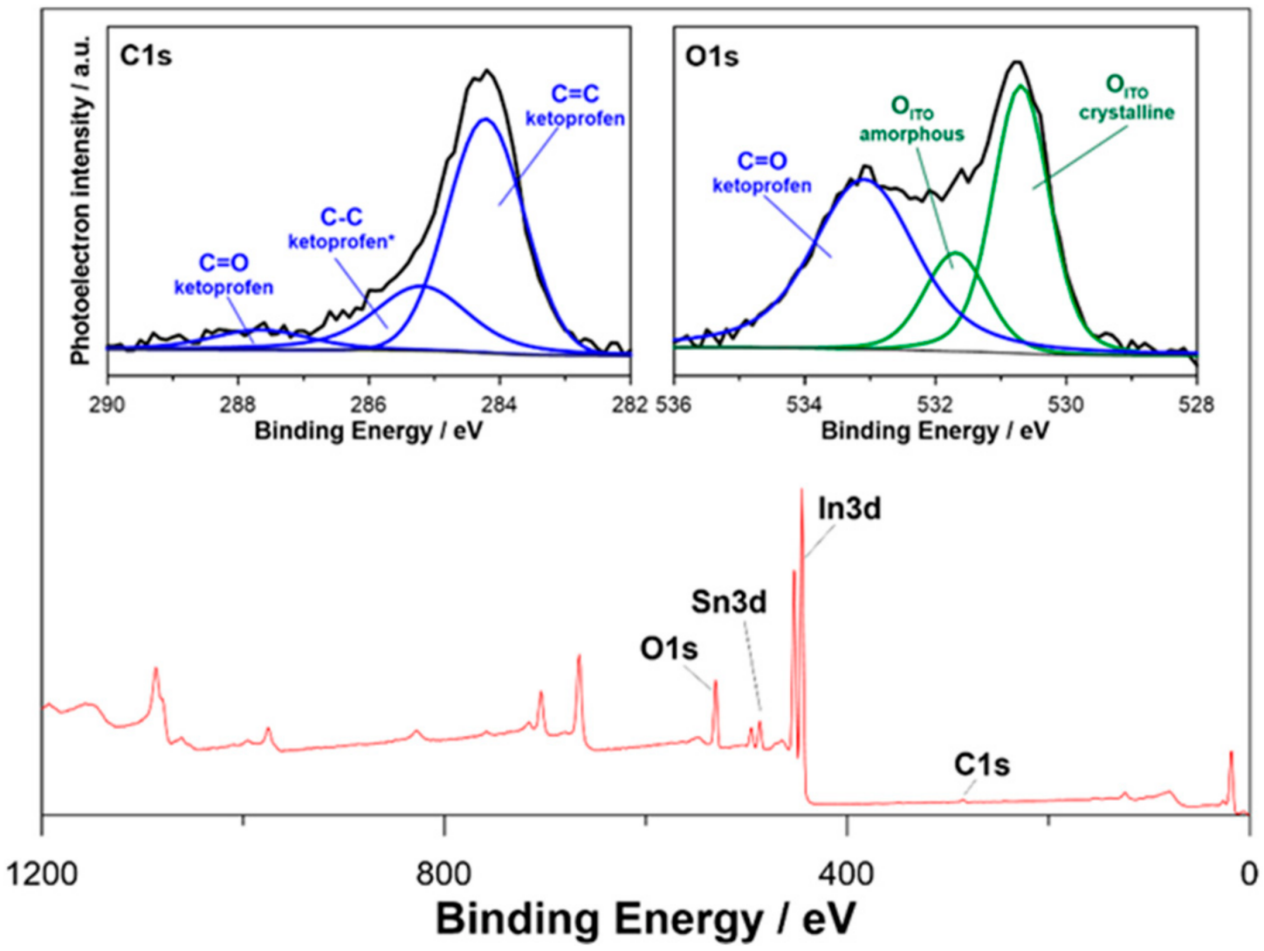
XPS survey spectrum and high-resolution XPS spectra registered for C1s and O1s energy range. Peaks underwent spectral deconvolution are superimposed with colors depending on their origination (blue for KP and green for ITO). The KP concentration was 1 × 10^−3^ M.

High-resolution XPS spectra, analyzed within the energy range of C1s and O1s peaks, make it possible to verify successful KP modification of ITO surface at the level of 10^−3^ M. Survey of the XPS spectrum presented in Figure 6 reveals significant contribution from ITO background seen as tin, indium, and oxygen peaks, and smaller contribution from electropolymerized thin KP layer on ITO surface. The high-resolution XPS spectra were also acquired, in the energy range characteristic for C1s and O1s peaks. The high-resolution analysis allows for verification of successful KP modification of ITO surface at the level of 10^−3^ M. Recorded spectra with their deconvolution are shown in the inset of Figure 6 and extracted data are summarized in Table 2.

The C1s spectrum was deconvoluted with three peaks, each denoting a different chemical state of carbon. The primary component is located at +284.2 eV and is characteristic for aromatic C=C bonds in KP. The second and third type of interaction can be associated with aliphatic C-C and C=O bonds. Their energy shift versus the primary spectral component is +1.0 for C–C and +3.5 eV for C=O type of bonds and highly correlates with other results found in the literature [41,42,43]. Furthermore, the XPS analysis carried out within the O1s energy region confirmed the pronounced presence of peak located at 533.1 eV, which is characteristic for carbonyl bonds. Finally, the acquired C=C:C–C:C=O ratio of 6.5:2.8:1 corresponds to the known for KP 6:1:1. A slight excess of C–C contribution can be explained by the presence of adventitious carbon coming from sample storage in atmospheric conditions. The amount of adventitious carbon found at bare ITO electrode did not exceed 5 at.% and was excluded from further analysis.

We have also performed detailed XPS analysis of peaks located in In3d5 and Sn3d5 energy range. The results of the analysis were also summarized in Table 2. According to literature survey, ITO analysis are typically based on spectral deconvolution using two sub-peaks—often ascribed to be contribution from crystalline and amorphous ITO. The observed peak shift between crystalline and amorphous phases—1.0 eV for In and 0.9 eV for Sn peaks—was found to stay in agreement with literature survey [44,45,46].

### 3.4. ITO-LMR-Based KP Electropolymerization Monitoring

**Figure 7:**
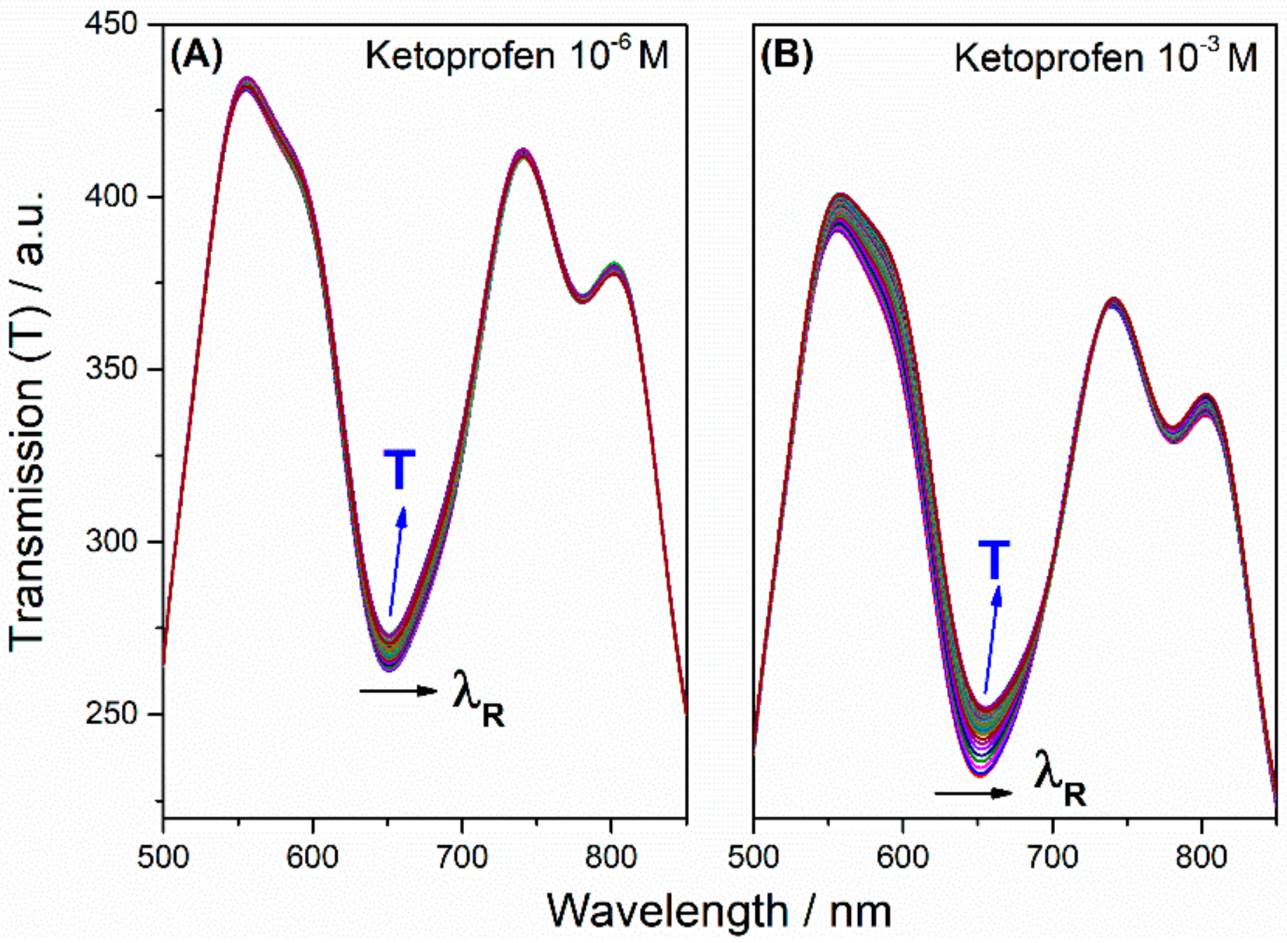
Changes in optical response of the ITO-LMR probe recorded during electropolymerization of KP on ITO surface for two KP concentrations, namely (**A**) 1 × 10^−6^ M and (**B**) 1 × 10^−3^ M.

The electrochemically-induced polymerization of KP was monitored optically using ITO-LMR probe. As shown in Figure 7, there were changes in the spectral response during cyclic voltammetry electropolymerization of KP for its two concentrations in the solution, i.e., the lowest and the highest. Obviously, for high KP concentration (1 × 10^−3^ M) results in electropolymerization of denser film which is followed by more pronounced changes in the optical spectrum (Figure 7B). Nevertheless, as low KP concentration as 1 × 10^−6^ M can be observed in optical response (Figure 7A). For all the applied concentrations, the most noticeable changes in the spectrum can be observed for the resonance at approx. λ_R_ ~650 nm, where a shift towards longer wavelengths takes place with the process progress. On top of tracking the resonant wavelength shift, in the discussed case also changes in *T* can be monitored at specific wavelength. For these resonance conditions, as previously when response to RI has been analyzed, we picked λ = 600 nm that is in the middle of the resonance slope. Due to the limited resolution of the spectrometer, T monitoring may deliver more accurate data than λ_R_.

The saturation of KP polymerization process was noticed for the ITO-LMR probe during the second CV cycle. The scan rate was set to 100 mV·s^−1^ in the range 0–2 V what resulted in 40 s per one cycle. The full range optical transmission was recorded with integration time up to 100 ms for 3500 data set. Thus, the entire optical analysis took 6 minutes. However, the wavelength range could be limited to e.g., 50 nm (approx. 250 data points) resulting in 25 seconds-long analysis. Summarizing, the result of KP determination was achieved with response time below 1 minute with no additional pretreatment, labeling, or incubation required.

**Figure 8:**
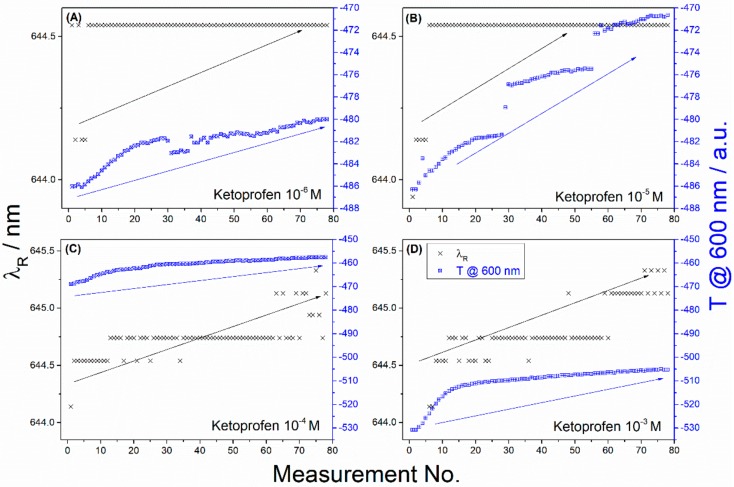
Change in resonance wavelengths (λ_R_) {×} and transmission (*T*) at 600 nm {□} with progress of KP electropolymerization process on ITO-LMR probe for KP concentration (**A**) 1 × 10^−6^ M; (**B**) 1 × 10^−5^ M; (**C**) 1 × 10^−4^ M; and (**D**) 1 × 10^−3^ M.

Variations of the two parameters, namely λ_R_ and *T* versus progress in the electrodeposition process are shown for all the KP concentrations in Figure 8. As in the case of an increase in external RI, both of them increase with progress of the electrodeposition process. The effect can be explained as a mass transfer of KP, i.e., densification of the medium at the ITO surface, resulting in the growth of the KP polymer film. A similar shift has already been reported for aptamer immobilization or swelling of poly-acrylic acid (PAA) and polyallylamine hydrochloride (PAH) polymeric coatings on ITO deposited on a fiber [26,47]. The increase in both parameters depends on the concentration of KP and surely has an impact on the thickness of the electrodeposited film. The effect of KP deposition on the electrode was revealed earlier by XPS studies (Figure 6) and recognized by a shift of peaks characteristic for aromatic C=C and aliphatic C–C and C=O bonds existing in this compound.

The relative changes of λ_R_ and *T* at λ = 600 nm are summarized in Table 3 using data shown in Figure 8. The final values of the achieved parameters after 80 measurements (approx. 120 s) were plotted versus the KP concentration. It must be noted that both the parameters linearly depend on the concentration with an average correlation coefficient *R*^2^ higher than 0.93. The sensitivities have been considered as ratios of the slopes reaching 1400.86 nm/M (nm·mg^−1^·L) and 16,422.46 a.u./M (a.u·mg^−1^·L) for resonance wavelengths (λ_R_) and *T*, respectively. The calculated LOD of KP is 0.536 or 0.575 mM using λ_R_ and *T*, respectively. The application of enhanced resolution equipment and standardized solutions at lower concentration would allow us to enhance this value as well as the LOD. The KP detection experiments were performed three times using separately deposited ITO-LMR probes. They were used to determine 1 mM KP solution with an average RSD value 8.5%, which indicates the satisfactory reproducibility and repeatability of the approach.

Analytical capability for KP determination with bare ITO-LMR electrode and other previously used nanomaterials is compared in Table 4. It can be concluded that the measurements with the ITO-LMR probe offer competitive sensitivity mainly when higher KP concentrations are considered. At these conditions standard electrochemical sensors are not effective due to adsorption at the electrode surface. The sensing concept can be further developed towards detection of other polymerizing agents. Fabrication of such sensors can be easily scaled-up keeping physical homogeneity and electrochemical performance. Summarizing, the application of the ITO-LMR probe offers competitive response toward KP detection mainly when larger concentrations are considered and standard electrochemical sensors are oversaturated by adsorption. The sensing concept can be further developed for future studies of other polymerizing agents.

## 4. Conclusions

In this study we have developed an optical fiber sensor based on the LMR effect supported by a thin ITO overlay and used it for real-time optical monitoring of electrochemical deposition of KP. The developed highly conductive ITO overlay was deposited on an optical fiber core and applied as a working electrode in cyclic voltammetry electrochemical setup. We have found that electrodeposition of KP on the ITO surface induces a significant change in the LMR response. The variation in optical transmission for the ITO-LMR sensor gradually follows the progress in the electrochemical deposition process. The sensor can be interrogated by tracing transmission at discrete wavelength as well as resonant wavelength shifts. Optical setup enables LMR monitoring of the KP concentration down to 1 × 10^−6^ M. Thus, the proposed method is a valuable alternative for the analysis of KP within the concentration range of 0.25–250 μg mL^−1^, allowing its determination at therapeutic and toxic levels. The sensing concept can be applied for detection of various other pharmaceuticals, as well as organics or biocompounds that are capable for electropolymerization at ITO surface. It is worth noting that this effect was obtained at bare ITO electrodes fabricated by magnetron sputtering. This deposition method is known for scalability and thus is widely applied as an industrial technology for a wide range of applications. The obtained devices are cheap in large-scale production, disposable, and can be applied in low-power, portable point-of-care devices or microchips. Moreover, the probes can be interrogated with simplified and limited in wavelength range systems based on LED source and Si photodiode with a bandpass filter.

## Figures and Tables

**Table 1:** Electrochemical parameters of the reactions for [Fe(CN)_6_]^3−/4−^ on the surface of bare and KP modified ITO electrodes.

Sample	E_red_ (mV)	E_ox_ (mV)	ΔE (mV)	E_1/2_ (mV)
Bare ITO electrode	−24	221	245	123
KP/ITO electrode	−230	281	511	230
Bare ITO-LMR electrode	−165	254	419	210
KP/ITO-LMR electrode	−285	343	628	314

**Table 2:** Comparison of chemical composition of bare ITO and ITO/KP electrode.

XPS Photopeak	Chemical State	Binding Energy (eV)	Chemical Composition (at.%)	
			Bare ITO Electrode	ITO/KP Electrode
C1s	C=C	284.2	-	27.8
	C–C *	285.2	-	9.9
	C=O	287.7	-	4.3
O1s	ITO_cryst_	530.7	40.5	13.6
	ITO_amorph_	531.7	12.8	5.5
	C=O	533.1	-	17.3
In	ITO_cryst_	444.1	29.8	9.8
	ITO_amorph_	445.1	11.8	9.2
Sn	ITO_cryst_	486.1	3.7	1.4
	ITO_amorph_	487.0	1.4	1.2

* Indicates the influence of adventitious carbon in total chemical composition of C–C chemical state.

**Table 3:** The relative changes of λ_R_ and *T* at 600 nm of ITO-LMR probe recorded vs. KP concentration.

KP Concentration	Δ*T* (a.u.)	Δλ (nm)
1 × 10^−3^ M	255.2	1.98
1 × 10^−4^ M	123.2	0.99
1 × 10^−5^ M	113.9	0.6
1 × 10^−6^ M	60.4	0.4

**Table 4:** Comparison of KP linear measurement range and LOD achieved with different methods.

Technique	Details	Linear Range	Limit of Detection	Reference
Adsorptive Stripping Square Wave	Mercury electrode	1 × 10^−8^–3 × 10^−7^ M	0.1 ng mL^−1^	[10]
LC-APCI-MS	Single Ion Monitoring mode (SIM)	100–500 ng/mL	1.0 ng/mL	[3]
IC-FLD	SnO_2_ nanoparticles	0.1 µg/kg	0.2–1.5 mg/kg	[5]
Differential Pulse Voltammetry	Aptamer and glassy carbon electrode	70 pM–6 µM	20 pM	[7]
Potentiometry	PVC electrode	0.0001–0.05 mol/L	6.3 × 10^−5^ mol/L	[11]
Microdialisys	Short polymeric columns (SPE)	25–5000 ng/mL	3 ng/mL	[48]
Flow injection	Flow injection with chemiluminescence	5.0 × 10^−8^–3.0 × 10^−6^ mol/L	2.0 × 10^−8^ mol/L	[6]
High-Performance Liquid Chromatography	Single-pass intestinal perfusion method	12.5–200 ng/mL	0.05 ng/mL	[49]
Rp-HPLC	PDA detector	872.5 nM	4.85–9.7 × 10^5^	[7]
Differential Pulse Polarography	Dropping-mercury electrode	1 × 10^−5^–5 × 10^−4^ M	9.8 × 10^−6^ mol/L	[8]
Polarography	Dropping-mercury electrode	10^−8^–10^−6^M	2.0 × 10^−9^ mol/L	[8]
Stripping voltammetry	Mercury electrode	1 × 10^−8^–1 × 10^−7^ M	2.0 × 10^−9^ mol/L	[9]
ITO-LMR probe	ITO electrode	1 × 10^−6^–1 × 10^−3^ M	0.5 × 10^−3^ mol/L	This work
